# Adolescent Loneliness and Negative Affect during the COVID-19 Pandemic: The Role of Extraversion and Neuroticism

**DOI:** 10.1007/s10964-023-01808-4

**Published:** 2023-06-30

**Authors:** Gabriela Gniewosz

**Affiliations:** Department of Education, University Innsbruck/Department of Education, Liebeneggstraße 8, A-6020 Innsbruck, Austria

**Keywords:** COVID-19, Loneliness, Negative affect, Personality traits, Longitudinal research

## Abstract

The COVID-19 pandemic had varied but significant effects on the lives of adolescents. This study aimed to examine the effects of extraversion and neuroticism on changes in loneliness and negative affect among adolescents during the pandemic. Longitudinal data were collected in three waves from 673 German adolescents and young adults (*M*_*age*_ = 16.8 years, SD_*age*_ = 0.91; female = 59%), affected by local lockdowns. The data collection was one time before (T1) and two times during the pandemic (T2, T3). Change score models were used to assess the relationship between loneliness and negative affect with consideration of extraversion and neuroticism. Results showed that pre-pandemic loneliness was predictive of changes in negative affect during the pandemic, with higher loneliness predicting increases in negative affect. Negative affect did not predict later loneliness. Extraverts showed an increase in negative affect over time, particularly between pre-pandemic measurement and the first phase of the pandemic. Higher neuroticism appeared to have increased vulnerability for negative affect during the pandemic, as a rise in negative affect were found among these adolescents throughout the course of the pandemic. In conclusion, the study highlights the significant impact of the COVID-19 pandemic on the mental health of adolescents and suggests that managing the pandemic during this specific developmental period is a challenge.

## Introduction

The transition from adolescence to young adulthood can be challenging as young people seek autonomy, identity formation, and stable relationships while facing puberty, school transitions, and post-school challenges (Branje et al., 2012; Branje et al., 2021). The COVID-19 pandemic added more difficulties for young people (≤18 years): Measures to stop the spread of the virus led to reduced social contact, likely increasing loneliness and negative emotions (Newlove-Delgado et al., 2022; Racine et al., 2021; Ravens-Sieberer et al., 2022). Although the pandemic was challenging for most adolescents, the pandemic affected adolescents differently, suggesting individual differences in vulnerability (Branje & Morris, 2021; Zager Kocjan et al., 2021). Socioeconomic (Bu et al., 2020), familial (Immel et al., 2022; Low & Mounts, 2022), and personality factors have been studied as possible risk factors for loneliness and negative affect (Choi et al., 2022; Cooper et al., 2021; Schmiedeberg & Thönnissen, 2021; López-Núñez et al., 2021). Previous research on personality traits during the pandemic has yielded mixed results, often lacking baseline measurements or focusing on broad age ranges (e.g., 14–70 years; Schmiedeberg & Thönnissen, 2021). This study aims to fill the gaps by investigating the relationship between adolescent loneliness and negative affect before and during the pandemic. Additionally, it examines whether extraversion and neuroticism can influence these factors and account for individual differences in vulnerability among adolescents and young adults.

### Loneliness and Negative Affect during Adolescence

A predominant way of defining loneliness sees it as a mismatch between the kinds of social relationships a person desires and those that they actually have (Peplau, 1985; see also, Von Soest et al., 2020). Loneliness differs from solitude, which refers to being alone by one’s own choice (Mund et al., 2019), and social isolation, which is the objective absence of close relationships (Laursen & Hartl, 2013; van Baarsen et al., 2001). Instead, loneliness refers to a subjective lack of close and meaningful relationships (Mund et al., 2019). As such, even individuals with similar degrees of actual social connection and sizes of support networks can differ in the extent to which they feel lonely (Houghton et al., 2016; Lasgaard et al., 2016).

Episodes of subjective loneliness are likely to affect individuals during the transition from adolescence to adulthood since this developmental stage involves (re-)establishing independent and egalitarian relationships with parents or other adults, engaging in romantic partnerships, and adapting to changes in social networks following graduation from school. Short-term periods of loneliness that are eventually resolved are unlikely to impose significant impairment or long-term consequences (Mund et al., 2019; Von Soest et al., 2020). However, for some adolescents, subjective loneliness becomes a persistent and pervasive burden. Being overwhelmed with various developmental tasks may cause feelings of losing social connection and stability, and the perception of being marginalized or cut off from others. Evidence shows that loneliness predicts deterioration in mental and physical health as well as elevated risk for early mortality (Courtin & Knapp, 2017; Dunn & Sicouri, 2022; Spithoven et al., 2017).

In the tripartite model of depression and anxiety, depressive symptoms are generally characterized by high negative and low positive affect (Clark & Watson, 1991). Increased negative affect during adolescence is associated with psychological maladjustment, perhaps being an indicator of ineffective adaptation to the changing demands and pressures of the social environment (Hanish et al., 2004; Young et al., 2019).

Much research has highlighted the interwoven and bidirectional relationship between loneliness and negative affect (Lasgaard et al., 2011; Spithoven et al., 2017; Vanhalst et al., 2012). The reduced quality and quantity of social relationships and support, an increased stress perception and rumination, as well as a reduced self-esteem and unpleasant subjective feelings of being alone may lead to increased and prolonged depressive symptoms, such as negative affect (Qualter et al., 2015; van Roekel et al., 2018). Depressed people, in turn, are generally less able to establish and engage in social contact with others or may often elicit rejection from interaction partners, for instance by excessive reassurance seeking, increasing the risk of loneliness (Vanhalst et al., 2012; Qualter et al., 2015; Qualter et al., 2010). Both loneliness and negative affect are conceptualized as subjectively unpleasant and emotionally distressing experiences (Cacioppo et al., 2006), and are both related to interpersonal problems, such as lack of support from close others (Spithoven et al., 2017).

However, there are notable differences between the two. Negative affect, but not loneliness, can occur in response to general difficulties, such as failure to adequately cope with negative or challenging situations (Lasgaard et al., 2011). Loneliness is often considered a specific form of emotional distress, whereas negative affect is considered to be a more general form (Cheng & Furnham, 2002; Spithoven et al., 2017). The developmental trajectories of loneliness and negative affect also differ during adolescence (12.1–17 years) and emerging adulthood (17.1–25 years), with feelings of loneliness typically peaking during middle adolescence, whereas negative affect continue to increase between adolescence and young adulthood (Mund et al., 2019; Nivard et al., 2015). Further, from early adolescence onward, females are affected by depressive symptoms such as negative affect significantly more often than males (Hankin & Abramson, 2001; Rutter, 2007; Zahn-Waxler et al., 2008), yet gender differences are not commonly observed for loneliness (Maes et al., 2019). As explained in more detail in the following section, it finally appears that personality traits such as extraversion and neuroticism may have varying effects on loneliness and negative affect, which suggests that these traits may contribute differently to individual differences in adolescents’ loneliness and negative affect.

### Relevance of Extraversion and Neuroticism

Personality traits such as extraversion and neuroticism are linked to interindividual differences in how situations are perceived and evaluated (Flesia et al., 2020; Horstmann et al., 2021; Schmiedeberg & Thönnissen, 2021; Vollrath, 2001). Depending on personal dispositions such as being more or less extraverted, individuals first evaluate whether a situation or event is relevant to their life and then focus on ways to deal with it (Lazarus & Folkman, 1987). In both appraisal and response, feelings of loneliness and negative affect may result. Numerous studies show that negative affect is correlated with both extraversion (negatively) and neuroticism (positively; Klimstra et al., 2010; Teppers et al., 2013; Vanhalst et al., 2012). Fewer studies, however, have examined associations between these traits and loneliness, though those that have suggest neuroticism and extraversion may be important correlates of loneliness (Cacioppo et al., 2006; Vanhalst et al., 2012).

The two traits differ, however, in how they affect negative affect and loneliness. These differences can be attributed to the fact that primary personality traits are associated with differences in individual’s subjective evaluations of emotional experiences (e.g., Larsen & Ketelaar, 1991; Verduyn & Brans, 2012). For instance, extraversion is related to positivity (e.g., joy, contentment, and satisfaction), and neuroticism to negativity (e.g., sadness, anxiety, and frustration). In this line, neuroticism represents a reduced capacity to effectively regulate negative emotions (Barańczuk, 2019; Ebstrup et al., 2011). Individuals high in neuroticism are more likely to perceive ambiguous situations as threatening, are more reactive to social stressors (Connor-Smith & Flachsbart, 2007; Vollrath, 2001), and more sensitive to possible social rejection (Denissen & Penke, 2008). Highly neurotic individuals are more likely to show dysfunctional interpersonal behavior, reducing their relationship satisfaction, social integration, and likability to others (Denissen & Penke, 2008; Vater & Schröder-Abé, 2015).

Extraversion, however, is the tendency to actively engage in social behaviors and enjoy social activities and interpersonal relationships (Mueller et al., 2019; Selfhout et al., 2010). Extraverts typically receive more support from others and experience positive interpersonal outcomes such as likeability and popularity (Asendorpf & Van Aken, 2003; Nikitin & Freund, 2015; van der Linden et al., 2010). Extraversion is also linked to a tendency to perceive challenging situations as manageable (Ebstrup et al., 2011), and to actually manage such situations more effectively through the use of appropriate emotion regulation and coping strategies (Barańczuk, 2019).

It is worth noting that loneliness and negative affect are not commonly understood to be facets of any personality characteristic such as neuroticism (Buecker & Horstmann, 2021; Mund et al., 2019), though loneliness and negative affect have been linked to the presence of negative and/or absence of positive affect. Extraversion and neuroticism may describe different mechanisms underpinning loneliness and negative affect over time. Although evidence on this matter is scarce, some longitudinal research shows that extraversion is more strongly related to loneliness than negative affect, whereas neuroticism shows the opposite pattern (Vanhalst et al., 2012). The link between loneliness and negative affect remains stable after controlling for personality traits, suggesting that the overlapping aspects of these phenomena can not be attributed to the same personality characteristics (Vanhalst et al., 2012).

### Neuroticism and Extraversion during the COVID-19 Pandemic

In Germany, the first nationwide lockdown was implemented between March and May 2020. Restrictions included the closure of schools and all other educational support services for adolescents, the prohibition of visits to playgrounds or shopping centers, and strict social distancing measures (Steinmetz et al., 2020). After the easing of the COVID-19 situation in May/June 2020, lockdown measures in Germany were continuously tightened and relaxed in response to increasing and falling cases. By the end of 2021, a total of four pandemic waves had occurred, each of which was met with contact restrictions and quarantine, remote work for most parents, and (partial) closure of schools and home-schooling. These restrictions were less strict than those of the first wave and allowed, for example, social contact in small groups outside the home or reduced schooling in classes with smaller groups (cf. Wechselunterricht).

The first lockdown implemented in March 2020 and subsequent lockdown measures created a stressful situation for most individuals and adolescents in particular (Flesia et al., 2020; Zager Kocjan et al., 2021). Adolescents as well as young and middle-aged adults (age range between 16 and 49 years) perceive and handle stressful situations differently according to their personality (Schmiedeberg & Thönnissen, 2021), so it seems reasonable to assume that adolescents with different levels of trait expression coped differently with the COVID-19 situation, both in the short and long term. Yet few studies have explicitly addressed adolescents’ change in negative affect and loneliness during the pandemic, or how personality differences might have influenced these changes. One study found extraverted adolescents (aged 14–17) to be more prone to negative changes in loneliness and negative affect than introverts during the early stages of the pandemic (Alt et al., 2021). Another study, which looked at 10–17-year-olds’ trajectories in internalization symptoms over four time points (two before and two after the first lockdown), confirmed increasing symptoms of loneliness and negative affect, yet also that friendship quality—a possible indicator for extraversion—was predictive of positive developments (Houghton et al., 2022). While not directly assessing personality traits, further research showed that in a sample of adolescents (15–17 years), poor pre-pandemic emotion regulation strategies—a defining aspect of neuroticism—were linked with increased depression and anxiety symptoms both during strict and relaxed contact restrictions (Breaux et al., 2021).

Bringing together the role of personality traits and adolescent problems associated with negative affect and loneliness during the pandemic, the results of higher increases in loneliness and negative affect experienced by neurotic individuals may be explained by their overall negative emotionality and tendency to react with maladaptive coping strategies to stressors (Barańczuk, 2019; Entringer & Gosling, 2021). Additionally, having problems in interpersonal relationships is more typical for neurotic people (Denissen et al., 2008) which makes it more difficult to maintain social contacts through periods of quarantine. It is likely that the pandemic reinforced dysfunctional interpersonal behaviors (Entringer & Gosling, 2021), perhaps causing increases in negative affect and loneliness.

In contrast, the increase in loneliness and decline in wellbeing (Hussong et al., 2021; Magson et al., 2021) attributed to extraverts during the first lockdown, reverses the typical pattern of results whereby extraversion is negatively associated with loneliness and mental health problems (Buecker & Horstmann, 2021). One explanation could be that extraverts, who seek social interaction more than others, are more affected by contact limitations. Specifically, extraverts seem to have an innate need for in-person social contact (Entringer & Gosling, 2021). A further explanation regards the effectiveness of extraverts’ coping strategies. For example, one strategy during the first weeks of the pandemic was to rely on social networks, such as planning face-to-face contact at school. Restrictions made these strategies ineffective (Swickert et al., 2002; Vollrath, 2001). Throughout the pandemic, however, it seems plausible that extraverts in particular were more able than others to use alternative and more successful coping strategies in the long run. Perhaps, extraversion may function as a protective factor (Nikčević et al., 2021) beyond the first weeks of the pandemic, because measures of social contact restrictions were increasingly relaxed and alternative strategies for making social contact were found.

## Current Study

Although current evidence indicates that the COVID-19 pandemic had an impact on adolescents’ social and emotional wellbeing, the understanding remains limited due to a scarcity of longitudinal studies assessing individual differences such as personality as possible explanatory factors. As previously outlined, few studies have examined whether extraversion and neuroticism affect changes in adolescent loneliness and negative affect over the course of the pandemic. The studies that do exist have used relatively small samples with a wide age range or relied upon a limited number of assessments. Here, it seems worthwhile to examine whether under the ongoing, specific conditions of the pandemic, personality traits are (or are not) protective factors for these outcomes. This study seeks to fill gaps in the existing literature on how extraversion and neuroticism may account for differences in adolescents’ and young adults’ mental health during the COVID-19 pandemic. Specifically, the study looks at the prediction of two different types of change in negative affect and loneliness: proportional change, which refers to time-dependent effects that may vary during different phases of the pandemic, and general changes or random effects, which represent individual differences that occur independently of time. By examining these different forms of change, the study aims to provide a more comprehensive understanding of how personality traits and the pandemic interact to affect adolescent wellbeing. To accomplish this, the study analyzes data from a nationally representative sample of German adolescents and young adults, collected at three different time points: before the pandemic (2019, time 1), during the first wave of COVID-19 (spring 2020, time 2), and 1 year later (spring 2021, time 3). This rationale informed the following hypotheses: Regarding changes between pre-pandemic (time 1) and the first wave of the pandemic (time 2), adolescents higher in extraversion will report increased loneliness and negative affect (Hypothesis 1). Further, adolescents higher in neuroticism will report an increase in loneliness and negative affect (Hypothesis 2). Regarding changes between the pandemic’s first wave (time 2) and 1 year later (time 3), those higher in extraversion will adjust better to the pandemic, showing no or lower increases in loneliness and negative affect (Hypothesis 3). In contrast, those higher in neuroticism will continue to show increases in loneliness and negative affect (Hypothesis 4). Regarding changes over the entire time, that is between time 1 and 3, it is expected that higher extraversion is associated with lower levels of loneliness and negative affect as well as little or no increases in either over time (Hypothesis 5). However, higher neuroticism is associated with higher levels of loneliness and negative affect as well as increases in both over time (Hypothesis 6).

## Method

### Procedure

This study is based on a dataset from the Panel Analysis of Intimate Relationships and Family Dynamics (pairfam) study conducted in Germany (Brüderl et al., 2020; Huinink et al., 2011), which comprises several cohorts and measurement points. Data for the present study refers to a cohort of adolescents and young adults born between 2001 and 03 (*N* = 1688) who were newly drawn in 2019 for Wave 11 (Cohort 4). Wave 11 (T1 in the current study) captured pre-pandemic information. A COVID-19 web survey in 2020 (T2) and wave 13 in 2020/21 (T3) captured data from the beginning and middle of the pandemic. T1 and T3 interviews were conducted face-to-face in participants’ homes using a computer-assisted personal interview (CAPI) as well as self-administered interviews (CASI). Interviews lasted approximately 60 m. T2 data was collected during the first wave of the pandemic between May 19th and July 13th 2020 (Walper et al., 2020). Participants were invited to participate in an online questionnaire taking ~15 m to complete.

However, to account for the strains experienced by adolescents during the COVID-19 pandemic, the data was restructured to include only individuals for whom information was available during the first wave of the pandemic (T2). The Table S4 outlines the sample selection process and dropouts over time (see, supplementary material). Initially, a total of *N* = 1688 individuals participated in wave 11 (T1), of which *n* = 855 were in middle adolescence and took part in the COVID-19 web-survey. Of these, 182 individuals were additionally excluded due to age outside the range of 15–18 years and incomplete interviews on all study variables from T1 to T3. Although there was a relatively high number of participants lost between T1 and T2, this was within the expected attrition rate for longitudinal studies, typically ranging between 30 and 70% (see, Benke et al., 2022; Luchetti et al., 2020). The decrease was attributed to a shift from personal interviews (CAPI, CATI) to online surveys and a shorter data collection period (see, Table S5 for further analysis in the supplemental material).

### Sample

The final sample comprised 673 adolescents, aged between 15 and 18 years (*M* = 16.82, SD = 0.91) who were predominantly have no migration background (79.0%). Female adolescents were slightly overrepresented (female = 59%; male = 41%). The majority of adolescents were in secondary school at T1 (88.4%) and attended the highest school track (Gymnasium: 61.8%). At T3, ~40.7% of adolescents were still in school, with 16.5% having started vocational training and 16.2% having enrolled in a university program. Most adolescents lived with their parents, before (98.8%) and during the pandemic (97.0%). A minority of the sample reported that they were poor which means that they and their families often have to forego something due to financial reasons (T1: 6.5%; T3: 5.5%). At T2, 27.7% reported a decrease in their families’ household income due to the pandemic, and 24.5% stated that the first wave of pandemic had strongly affected them personally in a negative way.

### Measures

All variables were assessed through adolescents’ subjective perspective. Table S1 (*see*, supplementary material) presents a detailed overview of items.

#### Negative affect

Negative affect was measured at all measurement points using a trait-scale of the State-Trait Depression Scales (STDS; Krohne et al., 2002). However, there were minor differences between T2 and the other time points, as the questions at T2 were presented in the past tense with reference to the first lockdown (e.g., T1/T3: “I feel sad” vs. T2: “I felt sad”). Negative affect was assessed through four items reflecting negative mood, including feelings of depression and sadness. Participants could respond from 1 (*almost never*) to 4 (*almost always*). The subscale had a good reliability at all measurement points (Guttman’s λ^2^_t1_ = 0.77, λ^2^_t2_ = 0.85, λ^2^_t3_ = 0.83).

#### Loneliness

At all measurement points, participants were asked to rate the extent to which they felt lonely (“I feel/ felt lonely”) with response option between 1 (*not at all*) and 5 (*absolutely*). As with negative affect, this item was presented differently at the second measurement point. This item is based on the UCLA Loneliness Scale (Russell et al., 1980). A single indicator for loneliness is typical in large-scale panel surveys (Mund et al., 2019; Mund et al., 2022; Pinquart & Sörensen, 2001) and has been found to be a sufficient method to capture loneliness as compared to multi-indicator assessments (see, Mund et al., 2022).

#### Extraversion & neuroticism

A well-established and widely used short version of the Big Five Inventory was used (BFI-S; Hahn et al., 2012; Rammstedt & John, 2005; Rammstedt & John, 2007) to assess extraversion and neuroticism. Adolescents were asked to rate how sociable and optimistic (e.g., “I get enthusiastic easily and can motivate others easily”) and how emotionally labile (e.g., “I worry a lot”) they generally perceived themselves. Response options ranged from 1 (*absolutely incorrect*) to 5 (*absolutely correct*). Extraversion and neuroticism were only assessed at T1 (Hahn et al., 2012; Rammstedt & John, 2007). Reliability for both measures was good (extraversion: Guttman’s λ^2^_t1_ = 0.80; neuroticism: Guttman’s λ^2^_t1_ = 0.70).

#### Covariates

Several covariates were included. Age, which ranged from 15 to 18 at T1 was considered to control for age-specific developmental differences. Gender (1 = male, 2 = female) served as a control variable, due to the robust finding that female adolescents are more prone to emotional problems than males (Dekker et al., 2007; Rutter, 2007). Migration background was included as a further control variable. A dichotomized variable was used, coded as 1 = no migration background and 2 = migration background, meaning that either the adolescents themselves or at least one parent or grandparent were not born in Germany (Barban & White, 2011). Information about adolescents’ school track at T1 (coded as 1 = low to 2 = high) was also included. Lower school tracks (Realschule, Hauptschule) primarily prepare for a vocational training, while the highest track (Gymnasium) leads to a maturity certificate (Abitur) and prepares students for university study or for a dual academic and vocational credential. Information about education at T3, coded categorically as 1 = in school, 2 = vocational training, and 3 = academic education, was included, as educational status changed for many between T1 (88.4% attended secondary school system) and T3 (40.07% attended secondary school system).

Table 1 shows descriptive statistics, reliability estimates, and correlations of all indicators. The MCAR test by Little (1988) was used to assess randomness of missing data in the analytical sample across three measurement points. The test was conducted using the R package MissMech (Jamshidian et al., 2014) and yielded a *p* value of 0.530, indicating random missingness.

**Table 1:** Means, standard deviations, reliability, and bivariate correlations

	Variable		M/ Freq.	SD/ %	λ^2^	1	2	3	4	5	6	7	8	9	10	11	12	13	14
1	Negative Affect T1		1.76	0.53	0.77														
2	Negative Affect T2		1.96	0.68	0.85	0.40**													
3	Negative Affect T3		1.88	0.59	0.83	0.54**	0.45**												
4	Loneliness T1		2.07	1.15	—	0.54**	0.31**	0.36**											
5	Loneliness T2		2.29	1.28	—	0.24**	0.52**	0.26**	0.27**										
6	Loneliness T3		2.30	1.17	—	0.37**	0.34**	0.56**	0.34**	0.37**									
7	Extraversion T1		3.35	0.93	0.80	−0.20**	0.02	−0.09*	−0.19**	−0.04	−0.10*								
8	Neuroticism T1		2.97	0.87	0.70	0.61**	0.34**	0.42**	0.38**	0.16**	0.24**	−0.28**							
9	Gender T1	m	*n* = 276	41.01%	—	0.17**	0.27**	0.22**	0.02	0.10*	0.08	0.11**	0.32**						
		f	*n* = 397	58.99%															
10	Age T1		16.82	0.91	—	−0.04	−0.03	−0.10*	−0.02	0.03	−0.05	0.05	−0.04	−0.01					
11	Migration Background T1	n	*n* = 532	79.05%	—	0.03	0.04	0.07	0.02	−0.03	0.01	−0.03	0.01	0.01	−0.01				
		y	*n* = 135	20.06%															
12	Highest School Track T1	l	*n* = 179	26.60%	—	−0.03	−0.02	0.01	0.03	0.05	0.01	0.13**	−0.01	0.02	0.24**	−0.09*			
		h	*n* = 416	61.81%															
13	School T3	n	*n* = 399	59.29%	—	−0.05	−0.06	0.04	−0.03	−0.06	−0.04	−0.02	−0.02	0.01	−0.39**	0.08	0.04		
		y	*n* = 274	40.71%															
14	Vocational Training T3	n	*n* = 560	83.21%	—	0.03	−0.05	−0.06	0.03	0.06	−0.01	0.01	0.03	−0.01	0.12**	−0.05	−0.20**	−0.37**	
		y	*n* = 113	16.79%															
15	Academic Education T3	n	*n* = 562	83.51%	—	−0.03	0.03	−0.06	0.05	0.00	0.03	0.05	−0.09*	−0.05	0.31**	−0.03	0.26**	−0.37**	−0.18**
		y	*n* = 111	16.49%															

*N* = 673; M/Freq and SD/% are used to represent mean/frequencies (for categorical data) and standard deviation/percent (for categorical data), respectively. λ^2^ represent Guttman’s reliability. All variables are reported by adolescents. T1 represents measurement point before COVID-19 pandemic; T2 and T3 represent measurement points during COVID-19 pandemic; Negative Affect represents one dimension of depressiveness; Gender: f = female & m = male; Migration Background: n = no migration background; y = person themselves/one parent/one grandparent not born in Germany; School Type: l = not highest secondary school track (Realschule or Hauptschule) & h = highest secondary school track (Gymnasium)

*p < 0.05; **p < 0.01; **p < 0.001

### Analytic Plan

Data and syntaxes that support the findings of this study are openly available in the Open Science Framework (Gniewosz, 2022). All analyses were conducted with the R package lavaan (Rosseel, 2012), using a robust maximum likelihood (ML) estimator (Finney & DiStefano, 2006). The full information ML adjustment method (Arbuckle et al., 1996) was applied to account for missing data. Additional fit indices were utilized to evaluate goodness of fit (e.g., RMSEA, TLI/CFI), but also present the *χ*^2^-test statistic (Hu & Bentler, 1999; Xia & Yang, 2019). Finally, 95% confidence intervals (95%-CI) were calculated.

Four steps were necessary to test the study’s hypotheses. At step one, confirmatory factor analysis and invariance over time were tested in separate models. For negative affect, invariance was specified on several consecutive and differently restricted models (Widaman et al., 2010). Differences between nested models, including configural (i.e., no parameter restrictions), weak (i.e., equal loadings), strong (i.e., equal loadings and intercepts) and strict (i.e., equal loadings, intercepts and residuals) invariant models, were tested for statistical significance using the difference of the *χ*^2^-values (Chen et al., 2001; Cheung & Rensvold, 2002). Based on strong invariant measurement models, composite scores (averages of the observed items for each scale) were used in the main analyses.

**Fig. 1:**
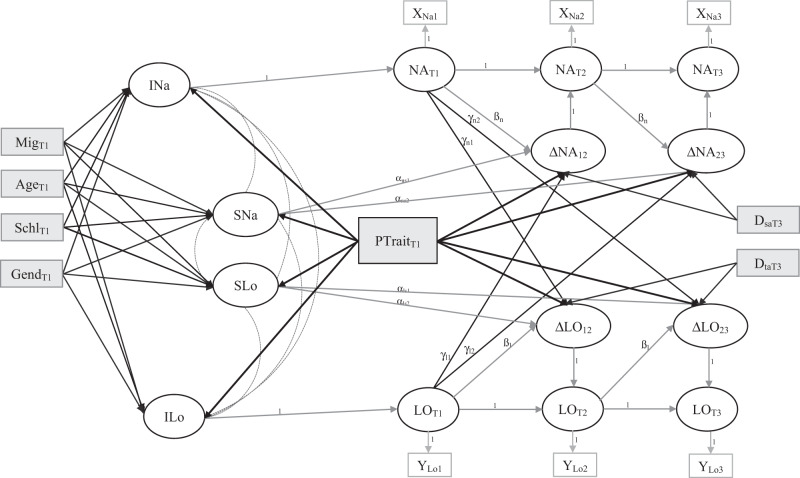
Schematic Model. Dual change score model with feedback and coupling parameters between loneliness (LO) and Negative Affect(NA) at three time points (T1–T3). Gray boxes represent manifest predictors and covariates; round circles represent latent variables; Age = Age T1; Schl = School Type T1 (low vs. high); Mig = Migration Background T1; Gend = Gender T1; D_saT3_ = Dummy education at T3 (school education vs. academic education); D_taT3_ = Dummy education at T3 (vocational training vs. academic education); PTrait = personality trait T1 (either neuroticism or extraversion); INa = General intercept negative affect; ILo = General intercept Loneliness; SNa = General slope negative affect; Slo = General slope loneliness; ΔNA_ij_ = Local Change aegative affect between two neighbored measurement points; ΔLO_ij_ = Local Change loneliness between two neighbored measurement points

At step two, dual Latent Change Score models were applied (LCSM, Kievit et al., 2018; Klopack & Wickrama, 2020; McArdle, 2009). This approach allows for simultaneous modelling of two latent variables (i.e., loneliness = LO and negative affect = NA) over time, while examining autoregressive feedback as well as coupling parameters (Fig. 1). Latent change should be differentiated into constant and proportional effects (Kievit et al., 2018). The constant effect (i.e., slope of NA = SNa; slope of LO = SLo) is a fixed parameter that represents general change across all time points and thus a measure of overall change. Proportional change refer to the change between two neighbored variables (i.e., change in NA between T1 and T2 = ΔNA_12_; change in LO between T1 and T2 = ΔLO_12_) and represents a more local change (cf. McHugh Power et al., 2019) of a variable relative to the previous state of that variable (Kievit et al., 2018). The latent change score is modeled by first measuring the variable at time 2 (i.e., NA at T2 = NA_T2_) with a factor loading fixed at 1, and then introducing a beta or feedback parameter to time 1 (i.e., βn). This allows for measurement of the impact of the variable at time 1 on the same variable at time 2. Feedback parameters were constrained equally over time, assuming the same feedback effects. Further, autoregressive paths between the two neighbored time points were fixed at 1. Perceived loneliness and negative affect were specified as latent indicator variables, and latent change scores were specified from scores at T1, with change modeled between T1 and T2, and between T2 and T3.

At step three, the effects of T1 loneliness on local changes of negative affect (ΔNA_12,_ ΔNA_23_), and T1 negative affect on local changes of loneliness (ΔLO_12,_ ΔLO_23_) were specified by introducing four additional coupling parameters. These represented time-dependent effects of one T1 variable on latent, local changes of the other (γ_n1_, γ_n2_, γ_l1_, γ_l2_). As a result, changes between T1 and T2 (i.e., ΔNA_12_) are a function of the constant (i.e., the slope of NA across the three time points = SNa) and proportional (i.e., NA at t1 = NA_T1_) effects as well as the value of the coupled variable at T1 (i.e., LO at t1 = LO_T1_). The changes between T2 and T3 (i.e., ΔNA_23_) are a result of the constant (i.e., SNa), the value of the same, previous measured variable (i.e., NA at t2 = NA_T2_) as well as the effect of the coupled variable at T1 (i.e., LO at T1 = LO_T1_).

At step four, the predictive value of extraversion and neuroticism as well as covariates were tested by adding these exogeneous variables into the dual LCSM model described above. Extraversion, neuroticism, age, gender, and migration background, were tested as predictors of general changes over time in loneliness and negative affect (i.e., SLO_,_ SNA), while extraversion, neuroticism, and education at T3 (specified as dummy variables: school vs. academic education and vocational training vs. academic education) were tested as predictors of local changes (i.e., ΔLO_12_ and ΔNA_12_ at T1 to T2; ΔLO_23_ and ΔNA_23_ at T2 to T3). An overview of the full LCSM is shown in Fig. 1.

## Results

### Pre-Analysis

First, invariance of negative affect over time were tested (T1 to T3) by applying the *χ*^2^-difference test. Strict invariance was found (see supplemental material, S-Table S2). The metric invariance was Δ*χ*^2^(Δ*df*) = 8.21(*6*), *p* = 0.223; strong invariance was Δ*χ*^2^(Δ*df*) = 2.89(*4*), *p* = 0.577; and strict invariance was Δ*χ*^2^(Δ*df*) = 8.52(*6*), *p* = 0.203. Based on this model, composite scores were used for the following models.

Next a descriptive model without prediction between latent variables (i.e., without coupling parameters of T1) and exogeneous variables (i.e., covariates, extraversion, neuroticism) were tested. This model showed an acceptable fit, *χ*^*2*^ (1, *n* = 673) = 1.46, *p* = 0.227; RMSEA = 0.026; CFI = 0.999; TLI = 0.992. Regarding general changes in loneliness (*M*_*SLo*_ = 1.56, *p* < 0.001) and negative affect (*M*_*SNa*_ = 1.46, *p* < 0.001) over time, participants reported an increase between T1 and T3. Regarding local changes, loneliness and negative affect increased between T1 and T2 (loneliness T1 – T2: *M*_*ΔLO12*_ = 0.78, *p* < 0.001; negative affect T1 – T2: *M*_*ΔNA12*_ = 0.76, *p* < 0.001), and between T2 and T3 (loneliness T2 – T3: *M*_*ΔLO23*_ = 0.79, *p* < 0.001; negative affect T2 – T3: *M*_*ΔNA23*_ = 0.70, *p* < 0.001). Increases between T1 and T2 and between T2 and T3 did not differ for loneliness (W*(1)* = 0.02, *p* = 0.876) or negative affect (W*(1)* = 3.43, *p* = 0.064).

Although mean values showed a negative trend over time, there were significant variances in general slopes and local change scores in loneliness and negative affect, pointing to interindividual differences over the entire time span as well as between the specific measurement time points. Latent means, mean differences, and variances are shown in Table 2.

**Table 2:** Latent means and changes: LCSM loneliness & negative affect

	Loneliness						Negative affect					
	Mean (Est.)	SE	*p value*	Variance (Est.)	SD	*p value*	Mean (Est.)	SE	*p value*	Variance (Est.)	SD	*p value*
General Intercept	2.07	0.04	0.001	1.32	1.15	<0.001	1.76	0.04	<0.001	0.29	0.53	<0.001
General Slope	1.56	0.09	0.001	0.61	0.78	<0.001	1.46	0.06	<0.001	0.27	0.52	<0.001
Local Change T1 - > T2	0.78	0.05	0.001	1.09	1.05	<0.001	0.76	0.04	<0.001	0.24	0.49	<0.001
Local Change T2 - > T3	0.79	0.06	0.001	0.76	0.87	<0.001	0.70	0.06	<0.001	0.13	0.36	<0.001

*N* = 673; Estimated Latent Means, Standard Error (SE), Variances and Standard Deviation (SD) are shown. Information is based on baseline bivariate LCSM model for negative affect and loneliness (no predictions); Robust Maximum Likelihood estimator was used, with Full Information Maximum Likelihood for handling missing data; t1 represents measurement point before COVID-19 pandemic; T2 and T3 represent measurement points during COVID-19 pandemic; Mean-difference in change scores loneliness T1–T2 vs. T2–T3: *W*(1) = 0.02, *p* = 0.876 & negative affect T1–T2 vs. T2–T3: *W*(1) = 3.43, *p* = 0.064; Variance-Difference in change scores loneliness T1–T2 vs. T2-T3: *W*(1) = 13.751, *p* < 0.001 & negative affect T1–T2 vs. T2-T3: *W*(1) = 10.23, *p* = 0.001; Model Fit for the bivariate LCSM model *χ*^*2*^(1) = 1.46, *p* = 0.227, RMSEA = 0.026, CFI/TLI = 0.999/0.992

### Main Analysis

The full LCSM model for negative affect and loneliness showed good model fit, *χ*^2^(*28*, *n* = 673) = 45.141, *p* = 0.021; RMSEA = 0.030; SRMR = 0.028; CFI = 0.989; TLI = 0.968. All information about the estimated parameters in the model are reported in Table 3. The latent correlation between extraversion and neuroticism was small with *r* = 0.310, S.E. = 0.03, *p* < 0.001.

**Table 3:** Bivariate LCSM negative affect & loneliness

	B	SE	β	*p* value	95%-CI	
*Regression Slopes*						
	*Change Loneliness T1 to T2*					
Loneliness T1 (feedback parameter)	**−0.960**	**0.067**	**−0.751**	**<0.001**	[**−**1.091;	**−**0.828]
Negative Affect T1	0.305	0.511	0.111	0.550	[**−**0.696;	1.307]
Neuroticism	0.027	0.078	0.016	0.724	[**−**0.125;	0.180]
Extraversion	0.016	0.027	0.010	0.549	[**−**0.037;	0.070]
School Type T1 (low vs. high)	0.121	0.114	0.038	0.287	[**−**0.102;	0.344]
	*Change Loneliness T2 to T3*					
Loneliness T2 (feedback parameter)	**−0.960**	**0.067**	**−0.905**	**<0.001**	[**−**1.091;	**−**0.828]
Negative Affect T1	0.456	0.494	0.178	0.356	[**−**0.512;	1.425]
Neuroticism	0.036	0.072	0.023	0.617	[**−**0.105;	0.178]
Extraversion	**−**0.020	0.026	**−**0.014	0.437	[**−**0.071;	0.031]
School Type T1 (low vs. high)	**−**0.006	0.111	**−**0.002	0.959	[**−**0.223;	0.211]
School T3 (school vs. academic)	**−**0.057	0.090	**−**0.021	0.525	[**−**0.234;	0.120]
Vocational Training T3 (vocational vs. academic)	**−**0.018	0.060	**−**0.010	0.765	[**−**0.135;	0.099]
	*Change Negative Affect T1 to T2*					
Negative Affect T1 (feedback parameter)	**−1.297**	**0.073**	**−0.927**	**<0.001**	[**−**1.440;	**−**1.155]
Loneliness T1	**0.298**	**0.071**	**0.506**	**<0.001**	[0.158;	0.437]
Neuroticism	**0.064**	**0.023**	**0.083**	**0.007**	[0.018;	0.110]
Extraversion	**0.028**	**0.012**	**0.039**	**0.014**	[0.006;	0.051]
School Type T1 (low vs. high)	**−**0.052	0.052	**−**0.035	0.326	[**−**0.154;	0.051]
	*Change Negative Affect T2 to T3*					
Negative Affect T2 (feedback parameter)	**−1.297**	**0.073**	**−0.913**	**<0.001**	[**−**1.440;	**−**1.155]
Loneliness T1	**0.223**	**0.063**	**0.385**	**<0.001**	[0.099;	0.347]
Neuroticism	**0.091**	**0.020**	**0.120**	**<0.001**	[0.051;	0.131]
Extraversion	0.018	0.012	0.025	0.137	[**−**0.006;	0.042]
School Type T1 (low vs. high)	0.015	0.055	0.010	0.784	[**−**0.092;	0.122]
School T3 (school vs. academic)	**0.110**	**0.044**	**0.081**	**0.013**	[0.023;	0.196]
Vocational Training T3 (vocational vs. academic)	**−**0.009	0.029	**−**0.010	0.749	[**−**0.066;	0.048]
	*General Intercept Loneliness*					
Neuroticism	**0.518**	**0.054**	**0.395**	**<0.001**	[0.411;	0.624]
Extraversion	**−0.101**	**0.048**	**−0.081**	**0.035**	[**−**0.194;	**−**0.007]
Age	0.002	0.044	0.002	0.956	[**−**0.085;	0.089]
Gender (male vs. female)	**−0.226**	**0.088**	**−0.097**	**0.010**	[**−**0.398;	**−**0.053]
Migration Background (no vs. yes)	0.020	0.096	0.007	0.833	[**−**0.167;	0.208]
	*General Slope Loneliness*					
Neuroticism	0.064	0.134	0.078	0.636	[**−**0.199;	0.326]
Extraversion	**−**0.004	0.030	**−**0.005	0.899	[**−**0.062;	0.055]
Age	**−**0.007	0.047	**−**0.009	0.883	[**−**0.098;	0.085]
Gender (male vs. female)	0.134	0.087	0.092	0.124	[**−**0.037;	0.304]
Migration Background (no vs. yes)	**−**0.034	0.103	**−**0.019	0.740	[**−**0.236;	0.167]
	*General Intercept Negative Affect*					
Neuroticism	**0.452**	**0.027**	**0.740**	**<0.001**	[0.400;	0.505]
Extraversion	**0.198**	**0.030**	**0.343**	**<0.001**	[0.140;	0.256]
Age	**−**0.010	0.017	**−**0.017	0.562	[**−**0.044;	0.024]
Gender (male vs. female)	**−0.072**	**0.036**	**−0.066**	**0.047**	[**−**0.143;	**−**0.001]
Migration Background (no vs. yes)	0.032	0.041	0.024	0.441	[**−**0.049;	0.113]
	*General Slope Negative Affect*					
Neuroticism	**0.155**	**0.037**	**0.269**	**<0.001**	[0.082;	0.228]
Extraversion	**0.046**	**0.016**	**0.085**	**0.004**	[0.015;	0.078]
Age	**−**0.023	0.025	**−**0.041	0.354	[**−**0.071;	0.025]
Gender (male vs. female)	**0.238**	**0.049**	**0.232**	**<0.001**	[0.143;	0.334]
Migration Background (no vs. yes)	0.093	0.053	0.074	0.081	[**−**0.011;	0.198]

*N* = 673; Unstandardized (B), standardized coefficients (β) as well as standard errors (SE) and *p* values are shown. Robust Maximum Likelihood estimator was used, with Full Information Maximum Likelihood for handling missing data. Negative affect represents negative affect. All variables were reported by adolescents. 95%-CI represents 95% Confidence Interval; T1 represents measurement points before COVID-19 pandemic; T2 and T3 represent measurement points during COVID-19 pandemic; Model Fit for the full bivariate LCSM model *χ*^*2*^(*28*) = 45.141, *p* = 0.021, RMSEA/SRMR = 0.030/0.028, CFI/ TLI = 0.989/0.96. Values in bold represent significant effects

Effects of control variables were on the whole negligible. For the proportional change in negative affect between T2 and T3, school attendance compared to academic education at T3 corresponded with a greater increase in negative affect (*B* = 0.11, *p* = 0.013). For general changes between T1 and T3, females showed stronger increases in negative affect over time (i.e., the general change or slope) than males (*B* = 0.24, *p* < 0.001). Females reported slightly lower levels of general loneliness (*B* = −0.23, *p* = 0.010) and negative affect than males (*B* = −0.07, *p* = 0.047). There were no other notable effects of control variables on general or local changes in loneliness or negative affect.

Regarding the relationship between loneliness and negative affect, only the coupling parameter from loneliness at T1 to local changes in negative affect was significant. Higher pre-pandemic loneliness was linked with greater increases in negative affect between T1 and T2 (*B* = 0.23, *p* < 0.001), and between T2 and T3 (*B* = 0.22, *p* < 0.001). However, negative affect at T1 was not significantly associated with changes in loneliness between T1 and T2 or between T2 and T3. Feedback parameters indicated that adolescents who had high scores in loneliness and negative affect at the previous time point reported a lower increase at the subsequent time point (for loneliness: *B* = −0.96, *p* < 0.001; for negative affect: *B* = −1.30, *p* < 0.001).

Regarding how neuroticism was linked to proportional changes in loneliness and negative affect, higher neuroticism was associated with greater increases in negative affect between T1 and T2 (*B* = 0.06, *p* = 0.007), and between T2 and T3 (*B* = 0.09, *p* < 0.001). Effects of neuroticism did not differ between T1 and T2 and between T2 and T3 (W*(1)* = 1.35, *p* = 0.245). During and beyond the first phase of COVID-19 pandemic, highly neurotic adolescents reported an increase of negative affect rather than feelings of loneliness. Regarding general levels and changes in loneliness and negative affect, higher neuroticism corresponded with higher general levels (general intercept) of loneliness (*B* = 0.52, *p* < 0.001) and negative affect (*B* = 0.45, *p* < 0.001). Further, adolescents reporting higher scores in neuroticism showed a stronger increase in general negative affect over time, but not loneliness (negative affect: *B* = 0.16, *p* < 0.001; loneliness: *B* = 0.06, *p* = 0.636).

Regarding how extraversion was associated with proportional changes in loneliness and negative affect, higher extraversion was only associated with stronger increases in negative affect between T1 and T2 (*B* = 0.03, *p* = 0.014). No other significant effects of extraversion on local changes in negative affect or loneliness were found^1^. Regarding effects of extraversion on adolescents’ general levels (between T1 and T3) and changes in loneliness and negative affect, participants higher in extraversion reported lower general loneliness (*B* = −0.10, *p* = 0.035), but higher overall negative affect (*B* = 0.20, *p* < 0.001). They also showed greater increases in general negative affect over time, but not loneliness (negative affect: *B* = 0.05, *p* = 0.004; loneliness: *B* = −0.01, *p* = 0.899).

### Sensitivity Analyses

To better understanding these results, subsequent analyses were conducted to test whether effects of extraversion and neuroticism on the change variables differed. Results are shown in Table S3 (supplemental material). The effects of neuroticism on the general intercepts of loneliness (W*(1)* = 108.51, *p* < 0.001) and negative affect (W*(1)* = 224.43, *p* < 0.001) were stronger than those of extraversion. Thus, adolescents with higher neuroticism scores reported higher general levels of loneliness and negative affect than those higher in extraversion. The effect of neuroticism on the general slope of negative affect was stronger than the effect of extraversion (W*(1)* = 14.75, *p* = 0.001), showing that adolescents with higher scores in neuroticism reported comparatively stronger increases in negative affect over the whole testing period. No differences between participants higher in extraversion and those higher in neuroticism were found for the general slope of loneliness (see, Table S3).

Difference tests on proportional changes indicated that the effect of neuroticism on negative affect between T2 and T3 was stronger than the effect of extraversion on change in negative affect between T2 and T3 (W*(1)* = 13.12, *p* = 0.001). No other significant differences between effects of neuroticism and extraversion were shown. Adolescents higher in neuroticism showed greater increases in negative affect than those higher in extraversion, but only for changes in negative affect between T2 and T3.

## Discussion

Managing the COVID-19 situation during the specific developmental period between late adolescence and early adulthood is a challenge for most young people (e.g., Racine et al., 2021; Ravens-Sieberer et al., 2022). Yet the pandemic did not affect all adolescents in the same way, suggesting that there may be individual differences in vulnerability (e.g., Immel et al., 2022; Low & Mounts, 2022). According to this perspective, the study investigates how extraversion and neuroticism traits affect loneliness and negative affect over time by using a three-wave longitudinal sample with one measurement point before and two measurement points during the pandemic. The associations over time were investigated in latent change score models (LCSM), helping to differentiate between proportional and general effects. The analysis included control variables such as adolescent age, gender, migration status, and school background. Subsequent analyses were conducted to test for potential differences in the effects of extraversion and neuroticism on the change variables.

Generally, this study found that pre-pandemic loneliness was substantially associated with changes in negative affect over the course of the pandemic, but not vice versa. In addition, extraversion and neuroticism predicted changes in negative affect, but not loneliness. For extraverted individuals, negative affect slightly increased over the entire time between T1 and T3 (Hypothesis 5 not confirmed) but particularly between the pre-pandemic measurement and the initial phase of the pandemic (Hypothesis 1 confirmed). Regarding changes during the pandemic (T2–T3) no associations emerged (Hypothesis 3 confirmed). Higher levels of neuroticism appear to increase vulnerability to negative affect during the pandemic. These adolescents showed an increase in negative affect during the entire course of the pandemic (Hypothesis 6 confirmed) as well as between pre-pandemic situation and the pandemic’s first wave (Hypothesis 2 confirmed) and continue to show increases in negative affect during pandemic’s first wave and 1 year later (Hypothesis 4 confirmed). Taken together, the results emphasize interindividual differences in the effects of the COVID-19 pandemic on adolescents’ and young adults’ mental health over time and highlights the importance of understanding risk and protective factors associated with these mental health outcomes. In the following, these results are discussed in more detail.

### Loneliness and Negative Affect

Analyses revealed large interindividual variance in changes of loneliness and negative affect, both over the entire time interval (T1–T3) and between individual time points (i.e., T1–T2; T2–T3). This indicates that young people coped with lockdowns differently, although increasing mean values in loneliness and negative affect indicate a pervasive burden of lockdowns on average. That said, even though in-person contact was limited, some adolescents appeared to have been more resilient. These individuals may have used more successful coping strategies to navigate through the pandemic, while others struggle to cope with an unpredictable and worrying situation (e.g., Branje & Morris, 2021; Orben et al., 2020).

The association between negative affect and loneliness in adolescents is well established cross-sectionally and longitudinally (Lasgaard et al., 2011; Spithoven et al., 2017; Qualter et al., 2013; van Roekel et al., 2018). Results showed that pre-pandemic loneliness was linked to increases in negative affect in both the early and later phases of the pandemic. Findings support the assumption that pre-pandemic loneliness increased the risk of poor mental health outcomes, but not vice versa (Houghton et al., 2022; Loades et al., 2020). It is possible that pre-pandemic loneliness increases vulnerability to experience psychological distress during difficult times (Pierce et al., 2020; Shanahan et al., 2022), leading to increased capacity for negative affect. Drawing from a personality psychology perspective, loneliness was expected to be a trait-like personality attribute, given that loneliness has found to be as stable over time as Big Five personality traits (Mund et al., 2019). Loneliness as a personality characteristic may affect perceptions and behaviors, especially under difficult conditions such as those experienced during COVID-19 restrictions.

The study’s findings further suggest that there are gender differences in the trajectory of negative affect over time, with females showing stronger increases than males. This is in line with previous findings on differences in the development of internal symptoms such as negative affect (e.g., Hankin & Abramson, 2001; Gomez-Baya et al., 2017; Pierce et al., 2020). These differences could be due to several factors, including differences in coping strategies, life circumstances, or hormonal changes. In contrast to previous research, females reported slightly lower levels of general loneliness and negative affect than males. The reasons for these differences are not clear from the underlying study alone. One reason may lie in the way the model is specified, i.e., the distinction between random and fixed effects. Thus, it may be that female adolescents between the ages of 15 and 18 are not generally more vulnerable than their male counterparts in this aspect but are more sensitive to (temporal) specific contextual changes (e.g., COVID-19 pandemic). However, the effects are very small and further research is needed to understand the complex relationships between gender and internal symptoms such as negative affect over time.

### The Role of Extraversion

In line with previous studies, evidence is provided that heightened extraversion can be a burden under certain conditions such as the social restrictions of the COVID-19 pandemic (Alt et al., 2021; Wijngaards et al., 2020; Zacher & Rudolph, 2021). Under typical conditions, the underlying findings support the protective effect of extraversion, as prior to the pandemic the trait was inversely correlated with negative affect (John et al., 2008). The specific conditions of the pandemic appeared to place highly extraverted adolescents under pressure, resulting in a more pronounced increase in negative affect compared to those lower in extraversion. It is likely that social isolation followed by repeated lockdowns caused particular difficulties for the more extraverted, who tend to thrive with face-to-face contact (Entringer & Gosling, 2021).

Results suggest also that those higher in extraversion to be vulnerable to negative long-term effects on wellbeing (T1–T3), as these individuals showed a greater increase in negative affect up to 1 year after the first phase of the pandemic. The rise in extraverts’ negative affect in the aftermath of the pandemic outbreak (T2) show that highly extraverted individuals did not regain their pre-pandemic level of wellbeing (T1) over time. One explanation for this may lie in the specificity of the pandemic situation, which differed from other disaster-like events in which higher extraversion has been shown to be a protective factor (Brock & Laifer, 2020; Gruber et al., 2021; Polizzi et al., 2020). Although the conditions of the pandemic enabled adolescents to engage in social contact through digital media, this type of interaction does not provide the level of close, in-person contact that seems fulfilling and necessary for extraverts’ wellbeing. Even a close family environment, known to play an important role in overcoming pandemic-related challenges (Brock & Laifer, 2020; Donker et al., 2021; Masten & Motti-Stefanidi, 2020), might not be able to fully compensate for extraverts’ need of real-life contact with friends and their extended support network.

However, the role of extraversion in explaining changes in negative affect may also more dynamic over time. While an increase in negative affect among extraverts between pre-pandemic assessment and the first wave of COVID-19 (T1–T2) was found, no such relationship was found for a subsequent phase of the pandemic, 1 year after the first lockdown (T2–T3). As the pandemic situation improved and restrictions relaxed, it might be found that extraverts in particular will recover more quickly to their pre-pandemic level of wellbeing. Future work could examine longer follow-up times to explore this possibility further.

Although some previous research had found higher extraversion to be associated with increases in loneliness (Alt et al., 2021; Romm et al., 2021), the underlying results suggest no higher increase in loneliness among extraverted youth (Luchetti et al., 2020). One explanation for this relates to how specific facets of extraversion might buffer against loneliness. For instance, as research under non-pandemic situations has shown, extraversion is linked to more positivity and likeability as well as popularity (Nikitin & Freund, 2015; van der Linden et al., 2010). These may buffer against loneliness but not compensate for the lack of real face-to-face contact associated with negative affect.

### The Role of Neuroticism

Previous research suggests a stronger link between neuroticism and negative affect than with loneliness (Vanhalst et al., 2012). The results here partly support this, and actually showed no association between neuroticism and loneliness. From these data, neuroticism is not associated with a rise in loneliness. However, inspecting correlations revealed that adolescents with high pre-pandemic neuroticism seemed to be vulnerable to loneliness. Neuroticism was positively associated with loneliness at T1, T2, and T3, which is in line with previous studies (John et al., 2008). Therefore, for individuals high in neuroticism, the pandemic situation is assumed to have only weak effects on increasing loneliness, which was already heightened.

Adolescents with higher neuroticism showed higher negative affect, with both a general increase over time (T1–T3) and an increase between specific measurement points (T1–T2 and T2–T3). This result is in line with other findings with adolescents (Breaux et al., 2021) and adults (Flesia et al., 2020; Nikčević et al., 2021; Schmiedeberg & Thönnissen, 2021). One explanation is that the pandemic increased unhealthy tendencies associated with higher neuroticism such as paying more attention to negative and threatening everyday stimuli or failing to respond effectively to stress, which could have added to feelings of negative affect (Ebstrup et al., 2011; Leger et al., 2016; Vollrath, 2001). A recent study that examined adolescent neuroticism and daily experiences provides evidence that neurotic individuals perceive everyday stimuli as more negative (Borghuis et al., 2020) than less neurotic individuals. The pandemic presented an additional challenge for neurotic adolescents, particularly given the dynamic and unpredictable nature of ongoing restrictions.

### Limitations

Despite the novelty and methodological strengths of the underlying study, some limitations should be kept in mind in interpreting the results. First, loneliness and negative affect were measured using brief self-reported assessments, and thus only specific aspects of these constructs could be tapped. Regarding negative affect, only negative affect was measured. However, lockdowns may have caused not only an increase in negative emotionality but also a reduction of positive affect (Clark & Watson, 1991). Loneliness was assessed with only a single item. Accordingly, reliability and validity of the measure may be suboptimal. However, a single indicator can be an adequate replacement for a larger scale and is widely used (Mund et al., 2019; Mund et al., 2022). Second, the study captures adolescents’ personal perceptions of negative affect and loneliness, and at the second measurement point this was only remembered negative affect. Retrospective assessment may show a recall-bias with recalled negative affect being worse than the actual experience (Urban et al., 2018). However, it could be expected that an immediate assessment of well-being in the first days/weeks of the pandemic would overlook negative changes, as they may develop with a delay. For instance, it was found that studies, assessing well-being immediately during the (first) lockdown mostly found no or only very small changes in well-being (Morales-Vives et al., 2020; Zager Kocjan et al., 2021), while studies with a longer time interval between (first) lockdown and assessment of well-being indicate negative developments (Alt et al., 2021; Zacher & Rudolph, 2021). Third, the study lacks information gathered from other family members that would help contextualize subjective information within a broader family system. For example, some prior research showed differences between adolescents’ and parents’ reports on family life and adolescent wellbeing during the COVID-19 pandemic (Cassinat et al., 2021; Li et al., 2021). Finally, there are likely more complex pathways of influence in which, for example, the links between personality and negative affect are mediated by feelings of loneliness, coping strategies, parental support, or other factors. For instance, research showed that a correlation between extraversion and negative affect was partially meditated by loneliness (Alt et al., 2021). Even more complex pathways, describing moderated mediation processes are also possible (see for example, Entringer & Gosling, 2021; Schemer et al., 2022).

## Conclusion

The COVID-19 pandemic has presented adolescents with an added challenge in terms of their mental health. Although studies recognize the significance of various social and individual factors influencing adolescents’ health and well-being during this difficult period, the current understanding remains limited due to a lack of longitudinal investigations examining individual differences through personality traits as potential explanatory factors. This study addressed the longitudinal relations between negative affect and loneliness by considering adolescent’s neuroticism and extraversion, using latent change score models (LCSM). The findings provide evidence that high levels of extraversion became burdensome for adolescents under the specific circumstances of social restrictions imposed in response to the COVID-19 pandemic. Additionally, adolescents with elevated levels of neuroticism already before the pandemic experienced the pandemic as an additional challenge, exhibiting increased negative affect throughout the pandemic. These results underscore the notion that the pandemic situation amplifies negative developments in already vulnerable adolescents and further highlight the struggle of previously low-risk individuals to effectively cope with the pandemic challenges, suggesting the emergence of new at-risk groups.

## Supplementary Information

Supplemental Tables

## Footnotes

1 Effects of extraversion did not differ between T1 and T2 and between T2 and T3 (W*(1)* = 0.34, *p* = 0.563).

## References

[CR1] Alt P, Reim J, Walper S (2021). Fall from grace: Increased loneliness and depressiveness among extraverted youth during the German COVID-19 lockdown. Journal of Research on Adolescence.

[CR2] Arbuckle, J. L., Marcoulides, G. A., & Schumacker, R. E. (1996). Full information estimation in the presence of incomplete data. In G. A. Marcoulides & R. E. Schumacker (Eds.), Advanced structural equation modeling: Issues and techniques (1st ed., Vol. 243, pp. 243–278). New York: Psychology Press.

[CR3] Asendorpf JB, Van Aken MAG (2003). Personality–relationship transaction in adolescence: Core versus surface personality characteristics. Journal of Personality.

[CR4] Barańczuk U (2019). The five factor model of personality and emotion regulation: A meta-analysis. Personality and Individual Differences.

[CR5] Barban N, White MJ (2011). Immigrants’ children’s transition to secondary school in Italy. International Migration Review.

[CR6] Benke, C., Autenrieth, L. K., Asselmann, E., & Pané-Farré, C. A. (2022). One year after the COVID-19 outbreak in Germany: long-term changes in depression, anxiety, loneliness, distress and life satisfaction. *European Archives of Psychiatry and Clinical Neuroscience*, 1–11. 10.1007/s00406-022-01400-0 10.1007/s00406-022-01400-0 PMC8962282 35348855

[CR7] Borghuis J, Bleidorn W, Sijtsma K, Branje S, Meeus WH, Denissen JJ (2020). Longitudinal associations between trait neuroticism and negative daily experiences in adolescence. Journal of Personality and Social Psychology.

[CR8] Branje, S., de Moor, E. L., Spitzer, J. & & Becht, A. I. (2021). Dynamics of identity development in adolescence: A decade in review. *Journal of Research on Adolescence, 31*(4), 908–927. 10.1111/jora.12678. 10.1111/jora.12678 PMC9298910 34820948

[CR9] Branje, S., Keijsers, L. G. M. T., Van Doorn, M. D., & Meeus, W. H. J. (2012). Interpersonal and intrapersonal processes in the development of adolescent relationships. In B. Laursen & W. A. Collins (Eds.), *Relationship pathways: from adolescence to young adulthood*. (pp. 257–276). Sage Publications Sage CA. 10.4135/9781452240565.n12

[CR10] Branje, S. & & Morris, A. S. (2021). The impact of the COVID-19 pandemic on adolescent emotional, social, and academic adjustment. *Journal of Research on Adolescence, 31*(3), 486–499. 10.1111/jora.12668. 10.1111/jora.12668 PMC8646893 34448306

[CR11] Breaux R, Dvorsky MR, Marsh NP, Green CD, Cash AR, Shroff DM, Buchen N, Langberg JM, Becker SP (2021). Prospective impact of COVID-19 on mental health functioning in adolescents with and without ADHD: protective role of emotion regulation abilities. Journal of Child Psychology and Psychiatry.

[CR12] Brock RL, Laifer LM (2020). Family science in the context of the COVID‐19 pandemic: Solutions and new directions. Family Process.

[CR13] Brüderl, J., Drobnič, S., Hank, K., Neyer, F. J., Walper, S., Alt, P., Bozoyan, C., Finn, C., Frister, R., Garrett, M., Gonzalez Avilés, T., Greischel, H., Gröpler, N., Hajek, K., Herzig, M., Huyer-May, B., Lenke, R., Minkus, L., Peter, T., Reim, J., Schmiedeberg, C., Schütze, P., Schumann, N., Thönnissen, C., Wetzel, M., & Wilhelm, B. (2020). *The German Family Panel (pairfam)*. 10.4232/pairfam.5678.11.0.0

[CR14] Bu F, Steptoe A, Fancourt D (2020). Who is lonely in lockdown? Cross-cohort analyses of predictors of loneliness before and during the COVID-19 pandemic. Public Health.

[CR15] Buecker S, Horstmann KT (2021). Loneliness and social isolation during the COVID-19 pandemic. European Psychologist.

[CR16] Cacioppo JT, Hawkley LC, Ernst JM, Burleson M, Berntson GG, Nouriani B, Spiegel D (2006). Loneliness within a nomological net: An evolutionary perspective. Journal of Research in Personality.

[CR18] Cacioppo JT, Hughes ME, Waite LJ, Hawkley LC, Thisted RA (2006). Loneliness as a specific risk factor for depressive symptoms: cross-sectional and longitudinal analyses. Psychology and Aging.

[CR19] Cassinat JR, Whiteman SD, Serang S, Dotterer AM, Mustillo SA, Maggs JL, Kelly BC (2021). Changes in family chaos and family relationships during the COVID-19 pandemic: Evidence from a longitudinal study. Developmental Psychology.

[CR20] Chen F, Bollen KA, Paxton P, Curran PJ, Kirby JB (2001). Improper solutions in structural equation models: Causes, consequences, and strategies. Sociological Methods & Research.

[CR21] Cheng H, Furnham A (2002). Personality, peer relations, and self‐confidence as predictors of happiness and loneliness. Journal of Adolescence.

[CR22] Cheung GW, Rensvold RB (2002). Evaluating goodness-of-fit indexes for testing measurement invariance. Structural Equation Modeling: A Multidisciplinary Journal.

[CR23] Choi J, Kim N, Kim J, Choi I (2022). Longitudinal examinations of changes in well-being during the early period of the COVID-19 pandemic: Testing the roles of extraversion and social distancing. Journal of Research in Personality.

[CR24] Clark LA, Watson D (1991). Tripartite model of anxiety and depression: Psychometric evidence and taxonomic implications. Journal of Abnormal Psychology.

[CR25] Connor-Smith JK, Flachsbart C (2007). Relations between personality and coping: a meta-analysis. Journal of Personality and Social Psychology.

[CR26] Cooper K, Hards E, Moltrecht B, Reynolds S, Shum A, McElroy E, Loades M (2021). Loneliness, social relationships, and mental health in adolescents during the COVID-19 pandemic. Journal of Affective Disorders.

[CR27] Courtin E, Knapp M (2017). Social isolation, loneliness and health in old age: a scoping review. Health & Social Care in the Community.

[CR28] Dekker MC, Ferdinand RF, Van Lang ND, Bongers IL, Van Der Ende J, Verhulst FC (2007). Developmental trajectories of depressive symptoms from early childhood to late adolescence: gender differences and adult outcome. Journal of Child Psychology and Psychiatry.

[CR29] Denissen JJ, Butalid L, Penke L, Van Aken MA (2008). The effects of weather on daily mood: a multilevel approach. Emotion.

[CR30] Denissen JJA, Penke L (2008). Neuroticism predicts reactions to cues of social inclusion. European Journal of Personality.

[CR31] Donker, M. H., Mastrotheodoros, S., & Branje, S. (2021). Development of parent-adolescent relationships during the COVID-19 pandemic: The role of stress and coping. *Preprint*. 10.1037/dev0001212 10.1037/dev0001212 34807684

[CR32] Dunn C, Sicouri G (2022). The relationship between loneliness and depressive symptoms in children and adolescents: A meta-analysis. Behaviour Change.

[CR33] Ebstrup JF, Eplov LF, Pisinger C, Jørgensen T (2011). Association between the Five Factor personality traits and perceived stress: is the effect mediated by general self-efficacy?. Anxiety, Stress & Coping.

[CR34] Entringer TM, Gosling SD (2021). Loneliness during a nationwide lockdown and the moderating effect of extroversion. Social Psychological and Personality Science.

[CR36] Finney, S. J., & DiStefano, C. (2006). Non-normal and categorical data in structural equation modeling. In G. R. Hancock & R. O. Mueller (Eds.), Structural equation modeling: A second course (pp. 269-314). Greenwich, CT: Information Age Publishing.

[CR37] Flesia L, Monaro M, Mazza C, Fietta V, Colicino E, Segatto B, Roma P (2020). Predicting perceived stress related to the Covid-19 outbreak through stable psychological traits and machine learning models. Journal of Clinical Medicine.

[CR38] Gniewosz, G. (2022). The Interplay of Loneliness and Negative Affect during Times of Covid-19 Pandemic: The Role of Adolescents‘ Extraversion and Neuroticism. 10.17605/OSF.IO/5UMGH 10.1007/s10964-023-01808-4 PMC10328868 37389714

[CR39] Gomez-Baya D, Mendoza R, Paino S, Gillham JE (2017). A two-year longitudinal study of gender differences in responses to positive affect and depressive symptoms during middle adolescence. Journal of Adolescence.

[CR40] Gruber J, Prinstein MJ, Clark LA, Rottenberg J, Abramowitz JS, Albano AM, Aldao A, Borelli JL, Chung T, Davila J (2021). Mental health and clinical psychological science in the time of COVID-19: Challenges, opportunities, and a call to action. American Psychologist.

[CR41] Hahn E, Gottschling J, Spinath FM (2012). Short measurements of personality–Validity and reliability of the GSOEP Big Five Inventory (BFI-S). Journal of Research in Personality.

[CR42] Hanish LD, Eisenberg N, Fabes RA, Spinrad TL, Ryan P, Schmidt S (2004). The expression and regulation of negative emotions: Risk factors for young children’s peer victimization. Development and Psychopathology.

[CR43] Hankin BL, Abramson LY (2001). Development of gender differences in depression: An elaborated cognitive vulnerability–transactional stress theory. Psychological Bulletin.

[CR44] Horstmann KT, Rauthmann JF, Sherman RA, Ziegler M (2021). Unveiling an exclusive link: Predicting behavior with personality, situation perception, and affect in a preregistered experience sampling study. Journal of Personality and Social Psychology.

[CR45] Houghton S, Hattie J, Carroll A, Wood L, Baffour B (2016). It hurts to be lonely! Loneliness and positive mental wellbeing in Australian rural and urban adolescents. Journal of Psychologists and Counsellors in Schools.

[CR46] Houghton S, Kyron M, Hunter SC, Lawrence D, Hattie J, Carroll A, Zadow C (2022). Adolescents’ longitudinal trajectories of mental health and loneliness: The impact of COVID-19 school closures. Journal of Adolescence.

[CR47] Hu L-T, Bentler PM (1999). Cutoff criteria for fit indexes in covariance structure analysis: Conventional criteria versus new alternatives. Structural Equation Modeling-a Multidisciplinary Journal.

[CR48] Huinink J, Brüderl J, Nauck B, Walper S, Castiglioni L, Feldhaus M (2011). Panel Analysis of Intimate Relationships and Family Dynamics (pairfam): Conceptual framework and design. Journal of Family Research.

[CR49] Hussong AM, Benner AD, Erdem G, Lansford JE, Makila LM, Petrie RC, The, S. R. A. C.-R. T. (2021). Adolescence amid a pandemic: Short- and long-term implications. Journal of Research on Adolescence.

[CR50] Immel L, Neumeier F, Peichl A (2022). The unequal consequences of the COVID-19 pandemic: Evidence from a large representative german population survey. Review of Income and Wealth.

[CR51] Jamshidian M, Jalal S, Jansen C (2014). MissMech: An R package for testing homoscedasticity, multivariate normality, and missing completely at random (MCAR). Journal of Statistical Software.

[CR52] John, O. P., Naumann, L. P., & Soto, C. J. (2008). Paradigm shift to the integrative big-five trait taxonomy: History, measurement, and conceptual issues. In O. P. John, R. W. Robins, & L. A. Pervin (Eds.), Handbook of personality: Theory and research (3rd ed., pp. 114–158). New York, NY: Guilford Press.

[CR53] Kievit RA, Brandmaier AM, Ziegler G, van Harmelen A-L, de Mooij SMM, Moutoussis M, Goodyer IM, Bullmore E, Jones PB, Fonagy P, Lindenberger U, Dolan RJ (2018). Developmental cognitive neuroscience using latent change score models: A tutorial and applications. Developmental Cognitive Neuroscience.

[CR54] Klimstra TA, Akse J, Hale WW, Raaijmakers QAW, Meeus WHJ (2010). Longitudinal associations between personality traits and problem behavior symptoms in adolescence. Journal of Research in Personality.

[CR55] Klopack ET, Wickrama K (2020). Modeling latent change score analysis and extensions in Mplus: A practical guide for researchers. Structural Equation Modeling.

[CR56] Krohne HW, Schmukle SC, Spaderna H, Spielberger CD (2002). The state-trait depression scales: an international comparison. Anxiety, Stress & Coping.

[CR57] Larsen RJ, Ketelaar T (1991). Personality and susceptibility to positive and negative emotional states. Journal of Personality and Social Psychology.

[CR58] Lasgaard M, Armour C, Bramsen RH, Goossens L (2016). Major life events as predictors of loneliness in adolescence. Journal of Child and Family Studies.

[CR59] Lasgaard M, Goossens L, Elklit A (2011). Loneliness, depressive symptomatology, and suicide ideation in adolescence: Cross-sectional and longitudinal analyses. Journal of Abnormal Child Psychology.

[CR60] Laursen B, Hartl AC (2013). Understanding loneliness during adolescence: Developmental changes that increase the risk of perceived social isolation. Journal of Adolescence.

[CR61] Lazarus RS, Folkman S (1987). Transactional theory and research on emotions and coping. European Journal of Personality.

[CR62] Leger KA, Charles ST, Turiano NA, Almeida DM (2016). Personality and stressor-related affect. Journal of Personality and Social Psychology.

[CR63] Li M, Li L, Wu F, Cao Y, Zhang H, Li X, Zou J, Guo Z, Kong L (2021). Perceived family adaptability and cohesion and depressive symptoms: A comparison of adolescents and parents during COVID-19 pandemic. Journal of Affective Disorders.

[CR64] Little RJ (1988). A test of missing completely at random for multivariate data with missing values. Journal of the American Statistical Association.

[CR65] Loades ME, Chatburn E, Higson-Sweeney N, Reynolds S, Shafran R, Brigden A, Linney C, McManus MN, Borwick C, Crawley E (2020). Rapid systematic review: the impact of social isolation and loneliness on the mental health of children and adolescents in the context of COVID-19. Journal of the American Academy of Child & Adolescent Psychiatry.

[CR66] López-Núñez MI, Díaz-Morales JF, Aparicio-García ME (2021). Individual differences, personality, social, family and work variables on mental health during COVID-19 outbreak in Spain. Personality and Individual Differences.

[CR67] Low N, Mounts NS (2022). Economic stress, parenting, and adolescents’ adjustment during the COVID-19 pandemic. Family Relations.

[CR68] Luchetti M, Lee JH, Aschwanden D, Sesker A, Strickhouser JE, Terracciano A, Sutin AR (2020). The trajectory of lonelines s in response to COVID-19. American Psychologist.

[CR69] Maes M, Nelemans SA, Danneel S, Fernández-Castilla B, Van den Noortgate W, Goossens L, Vanhalst J (2019). Loneliness and social anxiety across childhood and adolescence: Multilevel meta-analyses of cross-sectional and longitudinal associations. Developmental Psychology.

[CR70] Maes M, Qualter P, Vanhalst J, Van den Noortgate W, Goossens L (2019). Gender differences in loneliness across the lifespan: A meta–analysis. European Journal of Personality.

[CR71] Magson NR, Freeman JYA, Rapee RM, Richardson CE, Oar EL, Fardouly J (2021). Risk and protective factors for prospective changes in adolescent mental health during the COVID-19 pandemic. Journal of Youth and Adolescence.

[CR72] Masten AS, Motti-Stefanidi F (2020). Multisystem resilience for children and youth in disaster: Reflections in the context of COVID-19. Adversity and Resilience Science.

[CR73] McArdle JJ (2009). Latent variable modeling of differences and changes with longitudinal data. Annual Review of Psychology.

[CR122] McHugh Power JE, Steptoe A, Kee F, Lawlor BA (2019). Loneliness and social engagement in older adults: A bivariate dual change score analysis. Psychology and Aging.

[CR74] Morales-Vives F, Dueñas J-M, Vigil-Colet A, Camarero-Figuerola M (2020). Psychological variables related to adaptation to the COVID-19 lockdown in Spain. Frontiers in Psychology.

[CR75] Mueller S, Ram N, Conroy DE, Pincus AL, Gerstorf D, Wagner J (2019). Happy like a fish in water? The role of personality–situation fit for momentary happiness in social interactions across the adult lifespan. European Journal of Personality.

[CR76] Mund M, Freuding MM, Möbius K, Horn N, Neyer FJ (2019). The stability and change of loneliness across the life span: A meta-analysis of longitudinal studies. Personality and Social Psychology Review.

[CR77] Mund, M., Maes, M., Drewke, P. M., Gutzeit, A., Jaki, I., & Qualter, P. (2022). Would the real loneliness please stand up? The validity of loneliness scores and the reliability of single-item scores. *Assessment*, *4*. 10.1177/10731911221077227 10.1177/10731911221077227 PMC10149889 35246009

[CR78] Mund M, Neyer FJ (2014). Treating personality-relationship transactions with respect: narrow facets, advanced models, and extended time frames. Journal of Personality and Social Psychology.

[CR79] Newlove-Delgado, T., Russell, A. E., Mathews, F., Cross, L., Bryant, E., Gudka, R., Ukoumunne, O. C., & Ford, T. J. (2022). Annual research review: The impact of COVID-19 on psychopathology in children and young people worldwide: Systematic review of studies with pre- and within-pandemic data. *Journal of Child Psychology and Psychiatry*. 10.1111/jcpp.13716 10.1111/jcpp.13716 PMC10952503 36421049

[CR80] Nikčević AV, Marino C, Kolubinski DC, Leach D, Spada MM (2021). Modelling the contribution of the Big Five personality traits, health anxiety, and COVID-19 psychological distress to generalised anxiety and depressive symptoms during the COVID-19 pandemic. Journal of Affective Disorders.

[CR81] Nikitin J, Freund AM (2015). The indirect nature of social motives: The relation of social approach and avoidance motives with likeability via e xtraversion and a greeableness. Journal of Personality.

[CR82] Nivard MG, Dolan CV, Kendler KS, Kan KJ, Willemsen G, van Beijsterveldt CEM, Lindauer RJL, van Beek JHDA, Geels LM, Bartels M, Middeldorp CM, Boomsma DI (2015). Stability in symptoms of anxiety and depression as a function of genotype and environment: a longitudinal twin study from ages 3 to 63 years. Psychological Medicine.

[CR83] Orben A, Tomova L, Blakemore S-J (2020). The effects of social deprivation on adolescent development and mental health. The Lancet Child & Adolescent Health.

[CR84] Peplau, L. A. (1985). Loneliness research: Basic concepts and findings. In I. G. Sarason & B. R. Sarason (Eds.), *Social Support: Theory, Research and Applications* (pp. 269–286). Springer Netherlands. 10.1007/978-94-009-5115-0_15

[CR85] Pierce M, Hope H, Ford T, Hatch S, Hotopf M, John A, Kontopantelis E, Webb R, Wessely S, McManus S (2020). Mental health before and during the COVID-19 pandemic: A longitudinal probability sample survey of the UK population. The Lancet Psychiatry.

[CR86] Pinquart M, Sörensen S (2001). Gender differences in self-concept and psychological well-being in old age: A meta-analysis. The Journals of Gerontology Series B: Psychological Sciences and Social Sciences.

[CR87] Polizzi C, Lynn SJ, Perry A (2020). Stress and coping in the time of COVID-19: Pathways to resilience and recovery. Clinical Neuropsychiatry.

[CR88] Qualter P, Brown SL, Munn P (2010). Childhood loneliness as a predictor of adolescent depressive symptoms: an 8-year longitudinal study. Eur Child Adolesc Psychiatry.

[CR124] Qualter, P., Brown, S. L., Rotenberg, K. J., Vanhalst, J., Harris, R. A., Goossens, L., ... & Munn, P. (2013). Trajectories of loneliness during childhood and adolescence: Predictors and health outcomes. *Journal of Adolescence*, *36*(6), 1283–1293. 10.1016/j.adolescence.2013.01.005. 10.1016/j.adolescence.2013.01.005 23465384

[CR89] Qualter P, Vanhalst J, Harris R, Van Roekel E, Lodder G, Bangee M, Maes M, Verhagen M (2015). Loneliness across the life span. Perspectives on Psychological Science.

[CR90] Racine N, McArthur BA, Cooke JE, Eirich R, Zhu J, Madigan S (2021). Global prevalence of depressive and anxiety symptoms in children and adolescents during COVID-19: A meta-analysis. JAMA Pediatrics.

[CR91] Rammstedt B, John OP (2005). Kurzversion des Big Five Inventory (BFI-K): Entwicklung und Validierung eines ökonomischen Inventars zur Erfassung der fünf Faktoren der Persönlichkeit. Diagnostica.

[CR92] Rammstedt B, John OP (2007). Measuring personality in one minute or less: A 10-item short version of the Big Five Inventory in English and German. Journal of research in Personality.

[CR93] Ravens-Sieberer, U., Erhart, M., Devine, J., Gilbert, M., Reiss, F., Barkmann, C., Siegel, N. A., Simon, A. M., Hurrelmann, K., Schlack, R., Hölling, H., Wieler, L. H., & Kaman, A. (2022). Child and Adolescent Mental Health During the COVID-19 Pandemic: Results of the Three-Wave Longitudinal COPSY Study. *Journal of Adolescent Health*. 10.1016/j.jadohealth.2022.06.022 10.1016/j.jadohealth.2022.06.022 PMC9386895 35989235

[CR94] Romm KF, Park YW, Hughes JL, Gentzler AL (2021). Risk and protective factors for changes in adolescent psychosocial adjustment during COVID-19. Journal of Research on Adolescence.

[CR95] Rosseel Y (2012). lavaan: An R package for structural equation modeling. Journal of Statistical Software.

[CR96] Russell D, Peplau LA, Cutrona CE (1980). The revised UCLA Loneliness Scale: concurrent and discriminant validity evidence. Journal of Personality and Social Psychology.

[CR97] Rutter M (2007). Psychopathological development across adolescence. Journal of Youth and Adolescence.

[CR98] Schemer, L., Milde, C., Lischetzke, T., In-Albon, T., Karbach, J., Könen, T., & Glombiewski, J. A. (2022). Feeling lonely during the pandemic: Towards personality-tailored risk profiles. *Psychology, Health & Medicine*, 1–14. 10.1080/13548506.2022.2058030 10.1080/13548506.2022.2058030 35354349

[CR99] Schmiedeberg C, Thönnissen C (2021). Positive and negative perceptions of the COVID-19 pandemic: Does personality play a role. Social Science & Medicine.

[CR100] Selfhout M, Burk W, Branje S, Denissen J, Van Aken M, Meeus W (2010). Emerging late adolescent friendship networks and Big Five personality traits: A social network approach. Journal of Personality.

[CR101] Shanahan L, Steinhoff A, Bechtiger L, Murray AL, Nivette A, Hepp U, Ribeaud D, Eisner M (2022). Emotional distress in young adults during the COVID-19 pandemic: evidence of risk and resilience from a longitudinal cohort study. Psychological Medicine.

[CR102] Spithoven AWM, Lodder GMA, Goossens L, Bijttebier P, Bastin M, Verhagen M, Scholte RHJ (2017). Adolescents’ loneliness and depression associated with friendship experiences and well-being: A person-centered approach. Journal of Youth and Adolescence.

[CR121] Steinmetz, H., Batzdorfer, V., & Bosnjak, M. (2020). The ZPID lockdown measures dataset for Germany. ZPID Science Information Online *20* (1).

[CR103] Swickert RJ, Rosentreter CJ, Hittner JB, Mushrush JE (2002). Extraversion, social support processes, and stress. Personality and Individual Differences.

[CR104] Teppers E, Klimstra TA, Damme CV, Luyckx K, Vanhalst J, Goossens L (2013). Personality traits, loneliness, and attitudes toward aloneness in adolescence. Journal of Social and Personal Relationships.

[CR123] Urban EJ, Charles ST, Levine LJ, Almeida DM (2018). Depression history and memory bias for specific daily emotions. PloS one.

[CR105] van Baarsen B, Snijders TAB, Smit JH, van Duijn MAJ (2001). Lonely but not alone: Emotional isolation and social isolation as two distinct dimensions of loneliness in older people. Educational and Psychological Measurement.

[CR106] van der Linden D, Scholte RHJ, Cillessen AHN, Nijenhuis JT, Segers E (2010). Classroom ratings of likeability and popularity are related to the Big Five and the general factor of personality. Journal of Research in Personality.

[CR119] van Roekel E, Verhagen M, Engels RC, Scholte RH, Cacioppo S, Cacioppo JT (2018). Trait and state levels of loneliness in early and late adolescents: Examining the differential reactivity hypothesis. Journal of Clinical Child and Adolescent Psychology.

[CR107] Vanhalst J, Klimstra TA, Luyckx K, Scholte RHJ, Engels RCME, Goossens L (2012). The interplay of loneliness and depressive symptoms across adolescence: Exploring the role of personality traits. Journal of Youth and Adolescence.

[CR108] Vater A, Schröder-Abé M (2015). Explaining the link between personality and relationship satisfaction: Emotion regulation and interpersonal behaviour in conflict discussions. European Journal of Personality.

[CR109] Verduyn P, Brans K (2012). The relationship between extraversion, neuroticism and aspects of trait affect. Personality and Individual Differences.

[CR110] Vollrath M (2001). Personality and stress. Scandinavian Journal of Psychology.

[CR111] Von Soest T, Luhmann M, Gerstorf D (2020). The development of loneliness through adolescence and young adulthood: Its nature, correlates, and midlife outcomes. Developmental Psychology.

[CR112] Walper S, Sawatzki B, Alt P, Reim J, Schmiedeberg C, Thönnissen C, Wetzel M (2020). The pairfam COVID-19 survey. GESIS Datenarchiv, Köln. ZA5959 Datenfile Version.

[CR113] Widaman KF, Ferrer E, Conger RD (2010). Factorial invariance within longitudinal structural equation models: Measuring the same construct across time. Child Development Perspectives.

[CR114] Wijngaards I, Sisouw de Zilwa SC, Burger MJ (2020). Extraversion moderates the relationship between the stringency of COVID-19 protective measures and depressive symptoms. Frontiers in Psychology.

[CR115] Xia Y, Yang Y (2019). RMSEA, CFI, and TLI in structural equation modeling with ordered categorical data: The story they tell depends on the estimation methods. Behavior Research Methods.

[CR116] Young KS, Sandman CF, Craske MG (2019). Positive and negative emotion regulation in adolescence: links to anxiety and depression. Brain Sciences.

[CR117] Zacher H, Rudolph CW (2021). Big Five traits as predictors of perceived stressfulness of the COVID-19 pandemic. Personality and Individual Differences.

[CR118] Zager Kocjan G, Kavčič T, Avsec A (2021). Resilience matters: Explaining the association between personality and psychological functioning during the COVID-19 pandemic. International Journal of Clinical and Health Psychology.

[CR120] Zahn-Waxler C, Park J, Usher B, Belouad F, Cole P, Gruber R (2008). Young children’s representations of conflict and distress: A longitudinal study of boys and girls with disruptive behavior problems. Development and Psychopathology.

