# Supplementary material for: Adolescent Loneliness and Negative Affect during the COVID-19 Pandemic: The Role of Extraversion and Neuroticism
Source: J Youth Adolesc. 2023 Jun 30;52(9):1965–82. doi: 10.1007/s10964-023-01808-4 (PMC10328868; doi:10.1007/s10964-023-01808-4)
Supplement: Supplementary file 1 — Supplemental Tables [file 10964_2023_1808_MOESM1_ESM.docx]

**Supplemental Tables**

S-Table S1. Exact Item & Scale Information

| **Variable/ Scale** | **Nb. Item** | **Item Label** | **Values** |
| --- | --- | --- | --- |
| Negative Affect  (T1 to T3): | Item 1 (cor47i1 / per2i1) | My mood was melancholic | 1 = almost never to 4 = almost always |
|  | Item 2 (cor47i3 / per2i3) | I felt depressed | 1 = almost never to 4 = almost always |
|  | Item 3 (cor47i4 / per2i4) | I felt sad | 1 = almost never to 4 = almost always |
|  | Item 4 (cor47i6 / per2i6) | My mood was gloomy | 1 = almost never to 4 = almost always |
| Loneliness (T1 to T3) | Item 1(cor11i11 / per1i6) | I feel lonely | 1 = not at all to 5 = absolutely |
| Extraversion (T1) | Item 1 (per3i1) | I am usually modest and reserved (recoded) | 1 = absolutely incorrect to 5 = absolutely correct |
|  | Item 2 (per3i6) | I get enthusiastic easily and can motivate others easily | 1 = absolutely incorrect to 5 = absolutely correct |
|  | Item 3 (per3i11) | I tend to be the strong and silent type (recoded) | 1 = absolutely incorrect to 5 = absolutely correct |
|  | Item 4 (per3i16) | I am extroverted | 1 = absolutely incorrect to 5 = absolutely correct |
| Neuroticism (T1) | Item 1 (per3i4) | I easily become depressed or discouraged | 1 = absolutely incorrect to 5 = absolutely correct |
|  | Item 2 (per3i9) | I am relaxed and can handle stress well (recoded) | 1 = absolutely incorrect to 5 = absolutely correct |
|  | Item 3 (per3i14) | I worry a lot | 1 = absolutely incorrect to 5 = absolutely correct |
|  | Item 4 (per3i19) | I easily become nervous and insecure | 1 = absolutely incorrect to 5 = absolutely correct |
| Gender Item 1 (T1) | sex | Reported Gender | 1 = male to 2 = female |
| Age Item 1 (T1) | age | Age in years | 15 = 15 years to 18 = 18 years |
| Migration background (T1) | mig | The person themselves/ one parent/ one grandparent not born in Germany | 1 = no to 2 = yes |
| School Type (T1) | GymA11 | School Type | 1 = low/middle (Haupt-/ Realschule) to 2 = high (Gymnasium) |
| Secondary School (T3) | school | Dummy variable education at T3 (secondary school) | 1 = secondary school vs. 0 = other education |
| Vocational training/ education (T3) | training | Dummy variable education at T3 (vocational education) | 1 = vocational training vs. 0 = other education |

Note. Information originates from the pairfam scales manual (Brüderl et al., 2020; Walper et al., 2021)

S-Table S2. Longitudinal Measurement Invariance of Negative Affect

|  | **Fit Indices** | | | | | | | **𝜒^2^ - Difference Test** | | |
| --- | --- | --- | --- | --- | --- | --- | --- | --- | --- | --- |
|  | 𝜒^2^ | *df* | *p* | CFI | TLI | RMSEA | SRMR | Δ𝜒^2^ | *Δdf* | *p* |
| **Negative Affect (across T1, T2, & T3)** | | | | | | | |  |  |  |
| configural | 113.434 | 39 | <.001 | 0.969 | 0.948 | 0.061 | 0.035 |  |  |  |
| weak | 122.000 | 45 | <.001 | 0.968 | 0.953 | 0.057 | 0.038 | 8.21 | 6 | .223 |
| strong | 124.898 | 49 | <.001 | 0.969 | 0.958 | 0.055 | 0.039 | 2.89 | 4 | .577 |
| **strict^** | 133.566 | 55 | <.001 | 0.968 | 0.961 | 0.053 | 0.038 | 8.52 | 6 | .203 |

Note. N = 673. Robust Maximum Likelihood estimator was used, with Full Information Maximum Likelihood for handling missing data. CFI = Comparative Fit Index; TLI = Tucker-Lewis Index; RMSEA = Root Mean Square Error of Approximation; SRMR= Standardized Root Mean Square Residual; Model comparison was as follows: configural vs. weak model, weak model vs. strong model, strong model vs. strict model; ^Invariance Model (in bold) represents the best fitting model (= level of invariance).

S-Table S3. Effects of Extraversion and Neuroticism (pairwise comparison)

|  | **Regression Slopes** | | | **Difference Test (Wald-Test)** | | |
| --- | --- | --- | --- | --- | --- | --- |
|  | B | SE | *p* | W | *df* | *p* |
|  | **Change Loneliness T1 to T2** | | | | | |
| Neuroticism | 0.027 | 0.078 | 0.724 | 1.337 | 1 | .247 |
| Extraversion | 0.016 | 0.027 | 0.549 |  |  |  |
|  | **Change Loneliness T2 to T3** | | | | | |
| Neuroticism | 0.036 | 0.072 | 0.617 | 0.101 | 1 | .751 |
| Extraversion | -0.020 | 0.026 | 0.437 |  |  |  |
|  | **Change Negative Affect T1 to T2** | | | | | |
| Neuroticism | **0.064** | **0.023** | **0.007** | 2.732 | 1 | .098 |
| Extraversion | **0.028** | **0.012** | **0.014** |  |  |  |
|  | **Change Negative Affect T2 to T3** | | | | | |
| Neuroticism | **0.091** | **0.020** | **<0.001** | **13.124** | **1** | **.001** |
| Extraversion | 0.018 | 0.012 | 0.137 |  |  |  |
|  | **General Intercept Loneliness** | | | | | |
| Neuroticism | **0.518** | **0.054** | **<0.001** | **108.512** | **1** | **<.001** |
| Extraversion | **-0.101** | **0.048** | **0.035** |  |  |  |
|  | **General Slope Loneliness T1 to T3** | | | | | |
| Neuroticism | 0.064 | 0.134 | 0.636 | 1.166 | 1 | .280 |
| Extraversion | -0.004 | 0.030 | 0.899 |  |  |  |
|  | **General Intercept Negative Affect** | | | | | |
| Neuroticism | **0.452** | **0.027** | **<0.001** | **224.430** | **1** | **<.001** |
| Extraversion | **0.198** | **0.030** | **<0.001** |  |  |  |
|  | **General Slope Negative Affect T1 to T3** | | | | | |
| Neuroticism | **0.155** | **0.037** | **<0.001** | **14.746** | **1** | **.001** |
| Extraversion | **0.046** | **0.016** | **0.004** |  |  |  |

Note. N=673; Effects of extraversion and neuroticism (left hand side) as well as the test of difference between effects, using the Wald-Test are shown. The estimates based on the dual LCSM, presented in Table 3. The latent correlation between extraversion and neuroticism is *r* = .310, *S.E.* = .03, *p <* .001; 95%-CI [-0.375, -0.244]. Values in bold represent significant effects.

S-Table S4. Overview of Sample Size and Sample Reduction over Time

| **Sample (N)** | **Reduction (%)** | **Rationale** |
| --- | --- | --- |
| 1,688 | — | The youngest cohort born in 2001–2003 was added in wave 11 as a refreshment sample in the underlying panel data (pairfam-panel) |
| 855 | 49.34% | Of the youngest cohort, only 855 middle adolescents participated in the COVID-19 online survey (T2). The reasons are presumably the change in the data collection method (online survey instead of personal interviews) as well as a shortened data collection period. |
| 673 | 21.28% | Of the subsample of n=855, cases were additionally excluded due to an age outside the range of 15 to 18 years and predominantly incomplete interviews on all study variables over the entire time (T1 to T3). These individuals would not have contributed anything to the estimation, since not only individual items of some scales were missing, but the whole questionnaire was not completed. |

S-Table S5. Regression Model to predict Drop Out at T2

| **Predictor** | **B** | **B**  **95%-CI** | **β** | **β**  **95%-CI** | ***sr^2^*** | ***sr^2^***  **95%-CI** |
| --- | --- | --- | --- | --- | --- | --- |
| (Intercept) | 0.15 | [-0.19, 0.50] |  |  |  |  |
| School Track T1 | 0.13** | [0.09, 0.18] | 0.14 | [0.10, 0.19] | .02 | [.01, .03] |
| Migration Background T1 | -0.10** | [-0.14, -0.06] | -0.10 | [-0.14, -0.06] | .01 | [.00, .02] |
| Age T1 | 0.01 | [-0.02, 0.02] | 0.00 | [-0.04, 0.04] | .00 | [.00, .00] |
| Gender T1 | 0.11** | [0.07, 0.15] | 0.12 | [0.08, 0.17] | .01 | [.01, .03] |
| Fit | *R^2^*  = .051**; 95%-CI [.04,.07] | | | | | |

*Note.* Unstandardized (B), standardized coefficients (β) as well as semi-partial correlation squared (*sr^2^*) are shown. 95%-CI indicate the 95%- bootstrapping confidence interval (lower vs. upper limits of a confidence interval); Gender: 1 = female & 2 = male; Migration Background: 1 = no migration background; 2 = person themselves/ one parent/ one grandparent not born in Germany; School Type: 0 = not highest secondary school track (Realschule or Hauptschule) & 1 = highest secondary school track (Gymnasium); T1 represents measurement point before COVID-19 pandemic.

**p* < 0.05; ***p* < 0.01; ****p* < 0.01.
